# Utility of T_2_-weighted MRI texture analysis in assessment of peripheral zone prostate cancer aggressiveness: a single-arm, multicenter study

**DOI:** 10.1038/s41598-021-81272-x

**Published:** 2021-01-22

**Authors:** Gabriel A. Nketiah, Mattijs Elschot, Tom W. Scheenen, Marnix C. Maas, Tone F. Bathen, Kirsten M. Selnæs, The PCa-MAP Consortium, Ulrike I. Attenberger, Pascal A. T. Baltzer, Tone F. Bathen, Jurgen J. Fütterer, Masoom A. Haider, Thomas H. Helbich, Berthold Kiefer, Marnix C. Maas, Katarzyna J. Macura, Daniel J. A. Margolis, Anwar R. Padhani, Stephen H. Polanec, Marleen Praet, Tom W. Scheenen, Stefan O. Schoenberg, Kirsten M. Selnæs, Theodorus H. van der Kwast, Geert M. Villeirs, Trond Viset, Heninrich von Busch

**Affiliations:** 1 Department of Circulation and Medical Imaging, NTNU–Norwegian University of Science and Technology, Trondheim, Norway; 2 Department of Radiology and Nuclear Medicine, St. Olavs Hospital, Trondheim University Hospital, Trondheim, Norway; 3 Department of Radiology and Nuclear Medicine, Radboud University Medical Center, Nijmegen, The Netherlands; 4 Institute of Clinical Radiology and Nuclear Medicine, University Medical Center Mannheim, Mannheim, Germany; 5 Department of Biomedical Imaging and Image Guided Therapy, Medical University of Vienna, Vienna, Austria; 6 Department of Medical Imaging, University of Toronto, Lunenfeld Tanenbaum Research Institute, Sinai Health System, Ontario Institute of Cancer Research, Toronto, Canada; 7 Siemens Healthcare GmbH, MR Application Development, Erlangen, Germany; 8 The Russell H. Morgan Department of Radiology, The Johns Hopkins University, Baltimore, USA; 9 Prostate MRI and Abdominal Imaging Service, Weill Cornell Medicine, Weill Cornell Imaging, New York-Presbyterian, New York, USA; 10 Paul Strickland Scanner Center, Mount Vernon Cancer Center, London, UK; 11 Department of Radiology and Nuclear Medicine, Ghent University Hospital, Gent, Belgium; 12 Laboratory Medicine Program, Princess Margaret Cancer Center, University Health Network, Toronto, Canada; 13 Clinic of Laboratory Medicine, St. Olavs Hospital, Trondheim, Norway; 14 Siemens Healthcare GmbH, AI Products, Forchheim, Germany

**Keywords:** Cancer, Mathematics and computing

## Abstract

T_2_-weighted (T_2_W) MRI provides high spatial resolution and tissue-specific contrast, but it is predominantly used for qualitative evaluation of prostate anatomy and anomalies. This retrospective multicenter study evaluated the potential of T_2_W image-derived textural features for quantitative assessment of peripheral zone prostate cancer (PCa) aggressiveness. A standardized preoperative multiparametric MRI was performed on 87 PCa patients across 6 institutions. T_2_W intensity and apparent diffusion coefficient (ADC) histogram, and T_2_W textural features were computed from tumor volumes annotated based on whole-mount histology. Spearman correlations were used to evaluate association between textural features and PCa grade groups (i.e. 1–5). Feature utility in differentiating and classifying low-(grade group 1) vs. intermediate/high-(grade group ≥ 2) aggressive cancers was evaluated using Mann–Whitney U-tests, and a support vector machine classifier employing “hold-one-institution-out” cross-validation scheme, respectively. Textural features indicating image homogeneity and disorder/complexity correlated significantly (*p* < 0.05) with PCa grade groups. In the intermediate/high-aggressive cancers, textural homogeneity and disorder/complexity were significantly lower and higher, respectively, compared to the low-aggressive cancers. The mean classification accuracy across the centers was highest for the combined ADC and T_2_W intensity-textural features (84%) compared to ADC histogram (75%), T_2_W histogram (72%), T_2_W textural (72%) features alone or T_2_W histogram and texture (77%), T_2_W and ADC histogram (79%) combined. Texture analysis of T_2_W images provides quantitative information or features that are associated with peripheral zone PCa aggressiveness and can augment their classification.

## Introduction

Accurate assessment of localized prostate cancer aggressiveness is of utmost importance for determining patient treatment and follow-up strategies. Currently, this is determined based on Gleason and/or International Society of Urological Pathology (ISUP) grading^1,2^ of histological specimens, traditionally obtained by systematic transrectal ultrasound-guided biopsy sampling. Although the introduction of targeted approaches has improved biopsy sampling and cancer detection accuracies^3^, it is still limited to small portions of the prostate. With prostate cancer being a heterogeneous and multifocal disease, this can sometimes lead to inaccurate estimation of the disease extent, and thus undertreatment or overtreatment^4,5^. Moreover, biopsy sampling is invasive, and the risk of post-biopsy complications has become an increasing concern due to multidrug-resistance^6^. This makes repeated biopsy sampling unattractive in clinical practice, especially for active surveillance patients.

Multiparametric MRI (mpMRI) enables non-invasive acquisition of both anatomical [i.e. T_2_-weighted (T_2_W)] and functional [mainly diffusion-weighted (DW), and dynamic contrast-enhanced (DCE)] images of the entire prostate for cancer detection, staging, treatment planning, and response evaluation^7^. The introduction of mpMRI and the Prostate Imaging—Reporting and Data System (PI-RADS) guidelines^8^ have improved prostate cancer detection and accuracy^9^. DW and DCE MRI provide quantitative pathophysiological parameters such as apparent diffusion coefficient (ADC), and volume transfer constant (k^trans^) and extravascular-extracellular volume fraction (V_e_), which to some extent are capable of assessing prostate cancer aggressiveness^10,11^. Compared to DW and DCE, T_2_W MRI provides high spatial resolution and tissue-specific contrast, but currently, it is mainly used for qualitative evaluation of the prostate anatomy and anomalies.

Though important, qualitative assessment has several challenges and limitations, including dependency on subjective judgment of radiologists, which is prone to high inter-reader variability^12^ and the occurrence of equivocal findings in substantial number of cases^13^. Furthermore, with a multiparametric approach and the increasing availability of hybrid imaging modalities such as positron emission tomography/MRI^14^, the amount of data to be analyzed increases, making it also increasingly labor-intensive to manually collate all these images into meaningful information for clinical decision making.

Recently, radiomics, i.e. automatic high-throughput extraction of quantitative image features from radiological images and their subsequent analysis^15,16^, has gained attention with potential to overcome the above limitations and thus improve clinical decision making. Texture analysis constitutes a key methodology for extracting quantitative image features, particularly second- and high-order statistical image texture descriptors based on grey level co-occurrence matrix (GLCM)^17^ and grey level run length matrix (GLRLM)^18^, which examine spatial variations in pixel intensity distribution. Several interesting studies have reported the use of texture analysis in radiomics-based analysis of prostate cancer^19–23^, but have mostly been limited to single-center data. Previously, we^24^ showed that GLCM-based textural features derived from T_2_W images could potentially serve as non-invasive markers for assessing prostate cancer aggressiveness. Particularly, we found homogeneity and entropy features to correlate significantly with prostate cancer aggressiveness (i.e. grade groups 2 and 3) as defined on pathology, as well as with ADC and k^trans^. Also, the augmentation of quantitative MRI parameters with T_2_W image textural features enabled better classification. However, these preliminary findings were based on a relatively small number of patients recruited from single center. The aim of this current work was to validate and extend these findings using a multicenter cohort, and to investigate their performance in the classification of biopsy-proven prostate cancers.

## Materials and methods

### Patient population and data collection

The patient cohort data for this retrospective multicenter study constitutes part of a prospectively collected (between June 2010 and August 2015) data for the Prostate Cancer localization with a Multiparametric MR Approach trial (PCa-MAP; ClinicalTrials.gov Identifier NCT01138527)^25^. Eligible patients (N = 128) from six institutions: Johns Hopkins University, Baltimore (n = 20); Norwegian University of Science and Technology, Trondheim (n = 22); Radboud University Medical Centre, Nijmegen (n = 30); University of California, Los Angeles (n = 20); University Health Network, Toronto (n = 10); and Medical University of Vienna (n = 26) were included in this study. All patients were diagnosed with primary prostate cancer and were scheduled to undergo preoperative mpMRI with subsequent radical prostatectomy. The Regional Committee for Medical and Health Ethics (Mid Norway), the PCa-MAP trial consortium review board, as well the review board of each participating institution (HIPAA-compliant for USA institutions) approved this study and waived the requirement for written informed consent. All methods were performed in accordance with local institutional, national and international guidelines and regulations.

### MRI examination

The image acquisition protocol and/or settings were standardized across all centers, hence the term ‘single-arm’ in the title. All imaging was performed on 3T MRI systems (Siemens Healthineers) using standard vendor-supplied body and spine phased array coils for signal detection, without an endorectal coil. A minimum of four weeks was allowed between the last biopsy and MRI to avoid hemorrhage artifacts. The acquisition consisted of localizer scans, T_2_W, DW, DCE, and spectroscopic imaging. In this study, we utilized only the transverse T_2_W and DW images, which were acquired with a turbo spin-echo sequence (repetition/echo time (TR/TE): 4000/101 ms; field of view (FOV): 200 × 200 mm; matrix: 320 × 320; slice thickness: 3 mm; interslice gap: 0.6 mm), and a single-shot echo-planar sequence with four b-values: 0, 100, 400, and 800 s/mm^2^ (TR/TE: 3300/60 ms; FOV: 260 × 211 mm; matrix: 160 × 130; slice thickness: 3.6 mm), respectively. The images were oriented along the longest axis of the prostate, perpendicular to the urethra to best match routine histologic sectioning of the prostate. Pre-imaging preparations were performed in accordance with local institutional guidelines.

### Histopathologic examination and tumor delineation

Patients underwent radical prostatectomy within 12 weeks after the MRI examination. The prostatectomy specimens were prepared locally according to histopathology protocols at each institution, which included fixation, serial sectioning (perpendicular to the urethra to facilitate spatial matching to MRI) into ~ 3–4 mm axial slices, and hematoxylin and eosin staining of microsections. An experienced local uro-pathologist examined the stained slides, outlined cancer foci, described cancer location, and graded them in accordance with the Gleason scoring system^1,2^.

**Figure 1:**
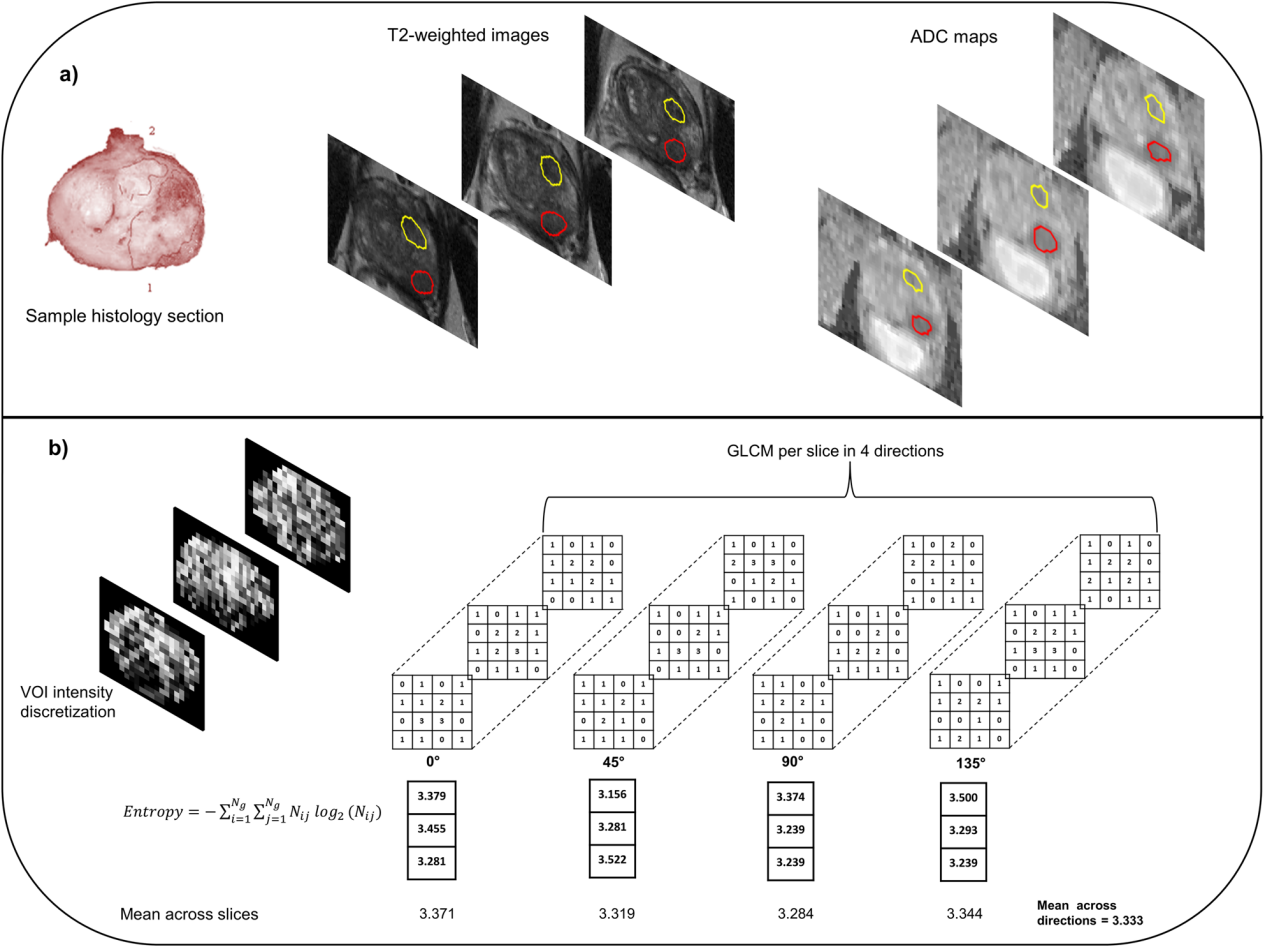
Illustration of tumor volume-of-interest (VOI) delineation and grey level co-occurrence matric (GLCM) texture feature extraction. (**a**) Histopathology slides were visually matched to the T_2_-weighted (T_2_W) images using anatomical landmarks. Peripheral (red) and transition (yellow) zone tumors were manually outlined on the T_2_W images based on their location on histology and appearance on T_2_W images, and then transformed to corresponding apparent diffusion coefficient (ADC) maps via registration or manually if the registration was deemed suboptimal. (**b**) Textural feature extraction from T_2_W VOIs using 2D average approach. For illustration purposes, the number of grey level bins was limited to four in this schematic, instead of the 32 used in actual computations.

The annotated whole-mount histology sections were visually matched to the T_2_W images based on anatomical landmarks such as urethra, ejaculatory ducts, size/shape of the peripheral zone and apex/base proximity. Moreover, descriptions from the pathology report were used as guidance. Tumor volumes of interest (VOIs) were then manually delineated (by Gabriel A. Nketiah, 5 years’ experience in prostate MRI; and guided by a radiologist Jurgen J. Fütterer with > 11 years’ experience) based on their location in histology and shape/appearance on the T_2_W images. The VOIs were subsequently transformed to the corresponding DW images via intensity-based rigid registration (Elastix toolbox^26^) using Mattes mutual information similarity metric (Fig. 1a). This was done by first co-registering the T_2_W images to the b = 0 s/mm^2^ images, and then applying the resulting transformation to the VOI masks. The co-registrations were visually verified, and manually corrected in case of mis-registration, for instance due to geometric distortion on the DW images. Each tumor was assigned a grade group (GG) according to the ISUP prostate cancer grading system^2^, and then dichotomized into low-(GG 1) and intermediate/high-(GG ≥ 2) aggressive cancers.

### Post-processing and feature extraction

Two types of features were computed from the image VOIs: traditional intensity histogram features (number of features, n_f_ = 11) from the T_2_W images and ADC maps, and second and high-order statistical image textural features (n_f_ = 29) based on GLCM^17^ and GLRLM^18^ from the T_2_W images. The “2D average” approach^27^ was employed to compute textural features from the tumor VOIs (Fig. 1b). First, the intensities within each VOI were discretized into 32 grey levels via fixed bin number quantization. The GLCMs and GLRLMs were computed per slice at one-pixel distance (*∂* = 1) in four symmetric directions, $\theta$ = 0°, 45°, 90° and 135°. Textural features were computed from each directional matrix, and the mean of each feature across the slices was obtained. Finally, the average of each feature across the four directions was calculated to eliminate potential differences in directionality. This 2D approach was preferred over 3D texture analysis due to the presence of interslice gaps in our data acquisition. ADC maps were calculated from the nonzero b-value DW image datasets (100, 400, and 800 s/mm^2^) by fitting a monoexponential decay model to the image intensities as a function of b-value in each voxel. ADC histogram features were then computed for each tumor VOI. The b = 0 s/mm^2^ image was excluded from ADC map computation to eliminate possible perfusion effects. The ADC was included as a benchmark metric for aggressiveness classification, since it has been previously shown to correlate with prostate cancer aggressiveness^10^.

Prior to feature extraction, the T_2_W images were corrected for intensity non-uniformity using the N4 bias field correction algorithm^28^, and subsequently normalized using the automated dual-reference tissue normalization approach^29^. Briefly, two aggregate feature channel object detectors were separately trained to detect fat and muscle tissue regions, from which reference intensity values (90th and 10th percentiles, respectively) were calculated, and then utilized to normalize the 3D image intensities to pseudo T2 values by linearly scaling the reference values to their corresponding T2 values at 3T from literature. Unlike T_2_W image intensities, the ADCs were not normalized because they are quantitative measurements in nature. Also, outlier voxels within each VOI, defined as intensities outside the range [µ − 3σ, µ + 3σ]^30^ were excluded; where μ and σ denote the mean and standard deviation of the intensities within each VOI. All features were computed in accordance with the image biomarker standardization initiative^27^.

### Statistical analysis and classification modeling

Spearman correlation coefficients were calculated to investigate associations between the T_2_W image features and prostate cancer grade groups (i.e. 1–5). Differences in feature values between the two aggressiveness classes (i.e. low versus intermediate/high) were evaluated using two-tailed Mann–Whitney U-tests. *p* values were corrected for multiple testing using Benjamini and Hochberg’s approach^31^ at false discovery rate of 0.05, with values < 0.05 considered statistically significant.

**Figure 2:**
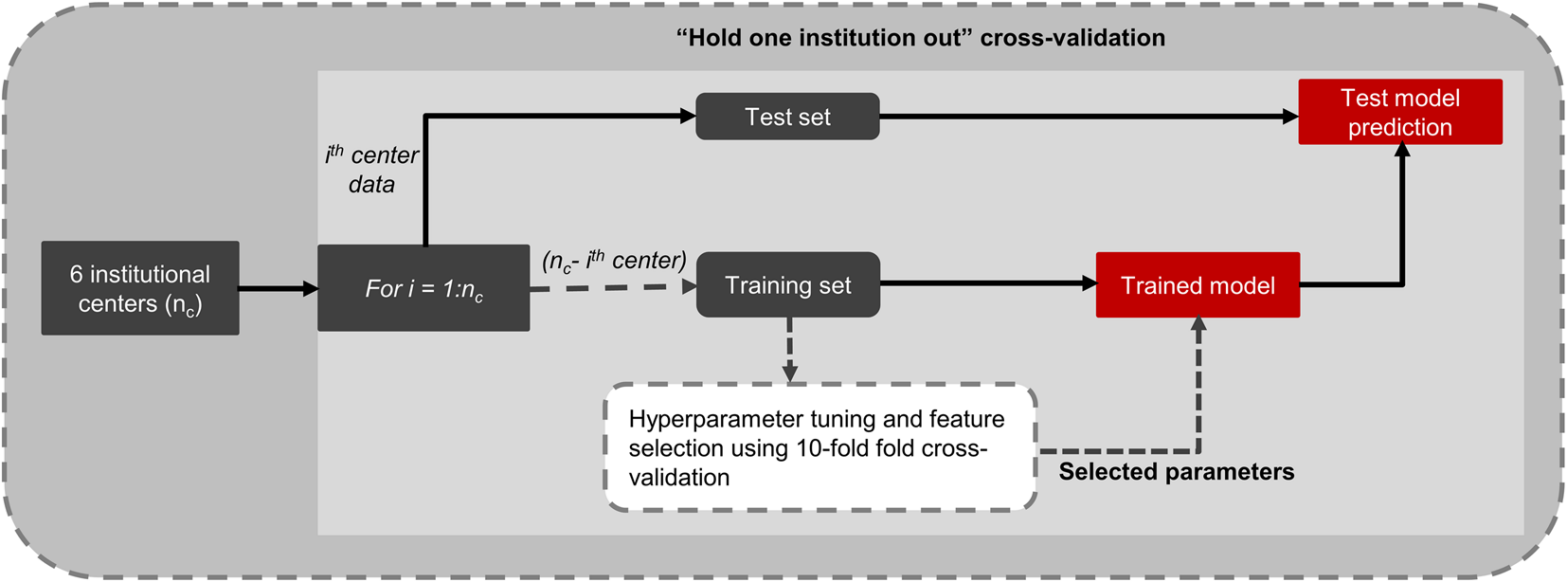
Schematic of the cross-validation scheme employed in support vector machine classifier training and testing across six institutional centers. At each iteration, data from one institution was held out for testing, and data from the remaining 5 institutions used for training.

To evaluate the utility of the features in classifying the two cancer aggressiveness classes, a linear support vector machine (SVM) classifier was trained and tested separately for each feature set (i.e. ADC histogram, T_2_W histogram and T_2_W textural features), and the following combinations: T_2_W histogram + textural features, ADC histogram + T_2_W histogram, and ADC histogram + T_2_W histogram + textural features. In this analysis, we were particularly interested in how well cancer aggressiveness at one institution could be predicted by a model trained on data from the other institutions, using histogram features with and without textural features augmentation. For this, the classifier was iteratively (*i* = *1:number of institutions, n*_*i*_) trained and tested, each time using data from *n*_*i*_ − *i* institutions as training set, with the *i*^*th*^ institution held out as an independent external test set (Fig. 2). The training employed stratified 10-fold cross-validation for hyperparameter tuning and feature selection. Hyperparameter (misclassification cost, C) tuning and feature selection during training were performed concurrently via grid search over seven logarithmically spaced values between − 1 and 1 inclusive, and using recursive feature elimination^32^, respectively. The hyperparameter and feature sets with the lowest mean misclassification error over all 10-folds were selected to build the model. The cross-validation partitioning of the data during training was done on patient level rather than on tumor level to ensure that multiple tumors from the same patient were all either in the training or in the validation subset. Predictions on the test set (i.e. data from the hold out institution) were however done on tumor-level.

Receiver operating characteristic (ROC) curves were computed for each test set, from which the area under the curve (AUC) and 95% confidence intervals (95CI), accuracy, sensitivity and specificity were calculated to evaluate the performances of the classifiers (i.e. feature sets) across the centers. The optimal threshold for calculating the accuracy, sensitivity and specificity was determined from the training based on the Youden index^33^, and then applied to the test set.

The added value of T_2_W textural features was evaluated using two approaches. First, at each institution, the AUCs before (i.e. ADC histogram, T_2_W histogram, and ADC + T_2_W histogram) and after augmentation with T_2_W textural features (i.e. T_2_W histogram + textural features, and ADC histogram + T_2_W histogram + textural features) were compared using Delong’s nonparametric approach for comparing the areas under two or more correlated receiver operating characteristic curves^34^. Secondly, the differences in performances across the institutions before and after augmentation with T_2_W textural features were compared using paired student t-test.

Prior to the classification modelling, two-way ANOVA was performed to evaluate potential effects of data origin (i.e. institution) and cancer aggressiveness on the features. Features for which the interaction between institution and cancer aggressiveness or main effect of institution were significant were excluded from the model. Each feature was log transformed to meet normality assumption requirement of ANOVA. The SVM classification modelling was performed with scikit-learn library^35^ in python (version 3.7, www.python.org), and other statistical analyses were performed in MATLAB R2019a (Mathworks).

## Results

**Figure 3:**
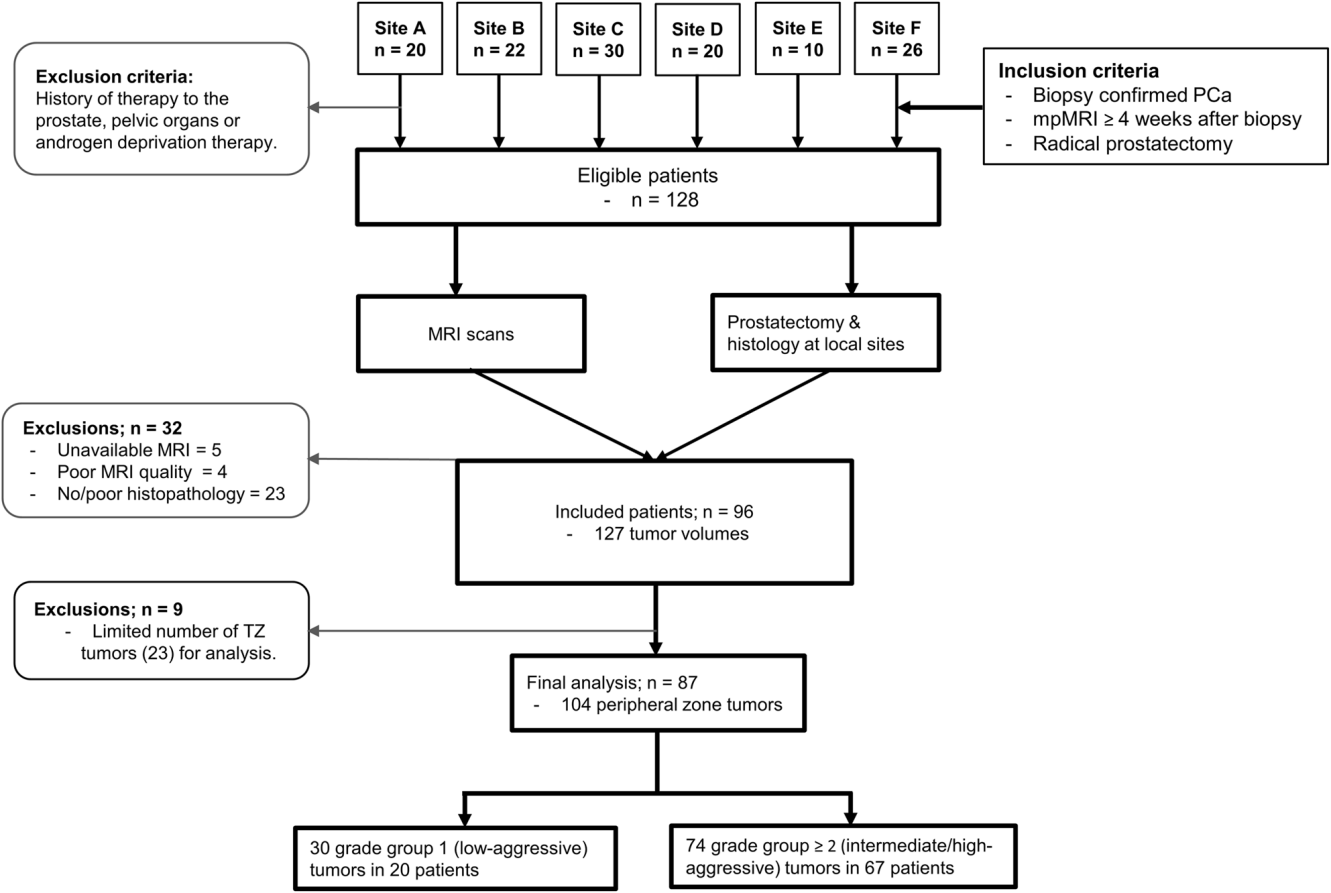
Flow chart of patients and datasets included in study. *mpMRI* multiparametric MRI, *PCa* prostate cancer, *TZ* transition zone.

Out of the 128 eligible patients, 32 patients were excluded due to unavailable MRI (n = 5), MRI artifacts and/or distortion (n = 4), no pathology report/grading (n = 15), unsatisfactory matching between histopathology and MRI (n = 8). Data of 96 patients (mean age = 61.3 ± 6.1 years) for whom good quality MRI and post-surgical histopathology data were available were included in this study. In these patients, 127 tumor volumes (mean [range] = 469 [101–1397] voxels) were identified, of which 104 were in the peripheral zone and 23 were in the transition zone. Figure 3 shows the flowchart of patient inclusion and exclusion. Due to the limited number of transition zone tumors, only the peripheral zone tumors (in 87 patients) were analyzed, of which 30 were stratified as low-aggressive, and 74 as intermediate/high-aggressive cancers. The overview of the characteristics of patients and tumors is given in Table 1.

**Table 1:** Characteristics of patients and tumors.

	Institutions						
	A	B	C	D	E	F	Overall
Number of patients	17	15	21	16	8	10	87
Age, years [range]	56 [46–70]	61 [52–73]	62 [51–69]	60 [55–69]	64 [50–71]	64 [52–76]	62 [46–76]
PSA ng/mL [range]	5.5 [1.7–18]	7.3 [1.9–11.2]	5.4 [2.5–20.8]	6.1 [3.6–12.5]	5.9 [3.1–17.3]	6.4 [5.2–31]	6.1 [1.7–31]
**Tumor grade group (aggressiveness class)**							
1 (L)	5	1	10	3	6	5	30
2 (I)	8	9	11	7	1	–	36
3 (H)	4	7	1	9	1	4	26
4 (H)	1	–	4	-	1	–	6
5 (H)	1	–	1	1	1	2	6
**Total**	19	17	27	20	10	11	104

*PSA* prostate specific antigen, *L* low, *I* intermediate, *H* high.

### Feature association with prostate cancer grade group

**Figure 4:**
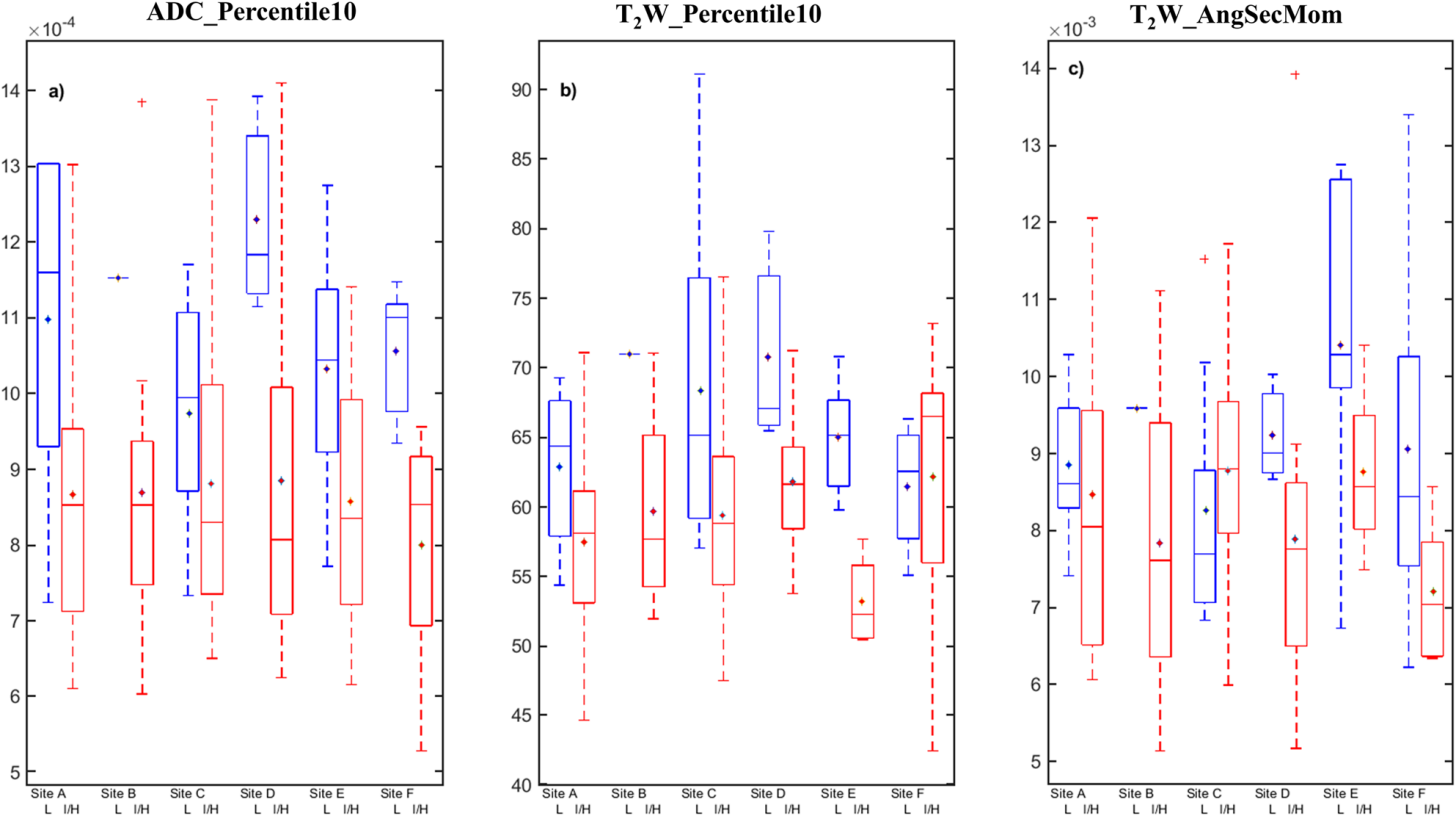
Box plots showing the distributions of apparent diffusion coefficient (ADC) and T_2_-weighted (T_2_W) histogram (10^th^ percentile), and T_2_W textural (angular second moment) features between low-aggressive (L) and intermediate/high-aggressive (I/H) prostate cancers at the participating institutions or sites. Notice that, where the T_2_W histogram feature (middle; Site F) overlap between the two cancer groups, an improved separation is observed for the textural feature (right; Site F), and vice-versa, indicating that the feature sets contain complementary information. *Indicates mean value.

The Spearman correlation between the image features and cancer grade groups was significant (*p* < 0.05) for eight T_2_W intensity histogram and nine textural features (Table 2). Differences in features between the two cancer aggressiveness classes were significant for seven intensity histogram and 16 textural features. Generally, the textural features reflected higher disorder/complexity (i.e. high entropy or lower homogeneity) in intermediate/high-aggressive tumors, and vice versa in low-aggressive tumors (Fig. 4). As expected, the majority of these features (7 intensity and 9 texture features) were found to be common between the two statistical tests.

**Table 2:** List of computed features from T_2_-weighted images and ADC maps, and their association with prostate cancer aggressiveness.

Feature type	List of computed features	Correlation (ρ) with cancer grade group		Differentiation of low versus I/H aggressive cancers: *p* values	
		*ADC*	T_2_W	*ADC*	T_2_W
Intensity histogram	Kurtosis	− 0.15	0.14	0.133	0.283
	Maximum	− 0.37*	− 0.23*	**0.015**	0.283
	Mean	− 0.45*	− 0.34*	**0.001**	**0.016**
	Median	− 0.44*	− 0.34*	**0.002**	**0.015**
	Minimum	− 0.50*	− 0.46*	**< 0.0001**	**< 0.0001**
	10th percentile	− 0.51*	− 0.39*	**< 0.0001**	**0.002**
	25th percentile	− 0.47*	− 0.35*	**0.001**	**0.006**
	75th percentile	− 0.41*	− 0.33*	**0.005**	**0.027**
	90th percentile	− 0.40*	− 0.31*	**0.006**	**0.048**
	Skewness	− 0.06	0.08	0.597	0.135
	Standard deviation	0.14	− 0.04	0.152	0.422
Textural (GLCM & GLRLM) features	ASM		− 0.34*		**0.043**
	Autocorrelation		− 0.02		0.597
	Cluster Prominence		− 0.19		**0.048**
	Cluster shade		0.02		0.422
	Contrast		− 0.18		**0.019**
	Correlation		0.03		0.597
	Difference entropy		− 0.09		0.084
	Difference variance		− 0.19		**0.019**
	Dissimilarity		− 0.17		**0.027**
	Entropy		0.38*		**0.028**
	IMC 1		0.51*		**< 0.0001**
	IMC 2		− 0.48*		**< 0.0001**
	IDM		0.13		**0.048**
	Maximum probability		− 0.32*		**0.048**
	Sum average		− 0.02		0.495
	Sum entropy		0.11		0.940
	Sum variance		− 0.23*		**0.027**
	Variance		− 0.25*		**0.012**
	Grey level non-uniformity		0.52*		**< 0.0001**
	HGLRE		− 0.06		0.364
	LGLRE		− 0.18		0.345
	Long run emphasis		0.17		**0.046**
	Long run high GLE		− 0.02		0.651
	Long run low GLE		− 0.14		0.557
	RLNU		0.50*		**0.001**
	Run percentage		− 0.17		**0.046**
	Short run emphasis		− 0.15		0.051
	Short run high GLE		− 0.07		0.293
	Short run low GLE		− 0.18		0.330

Bolded *p* values indicate features differed significantly between low- (grade group 1) and I/H-aggressive (grade group ≥ 2) prostate cancers.

*ASM* angular second moment, *IMC* information measure of correlation, *IDM* inverse difference moment, *HGLRE* high grey level run emphasis, *LGLRE* Low grey level run emphasis, *GLE* grey level emphasis, *RLNU* run length non-uniformity, *I/H* intermediate/high, *ρ* spearman correlation coefficient between features and cancer grade groups.

*Indicates feature correlated significantly with cancer grade group. The correlation coefficients were calculated for the entire grade groups (i.e. 1–5) and not for the dichotomized aggressive classes (i.e. grade groups 1 and ≥ 2).

The two-way ANOVA showed no statistically significant interaction between the effects of data origin (institution) and cancer aggressiveness on any feature. Similarly, the main effect of institution was not significant.

### Classification of low versus intermediate/high-aggressive cancers

**Figure 5:**
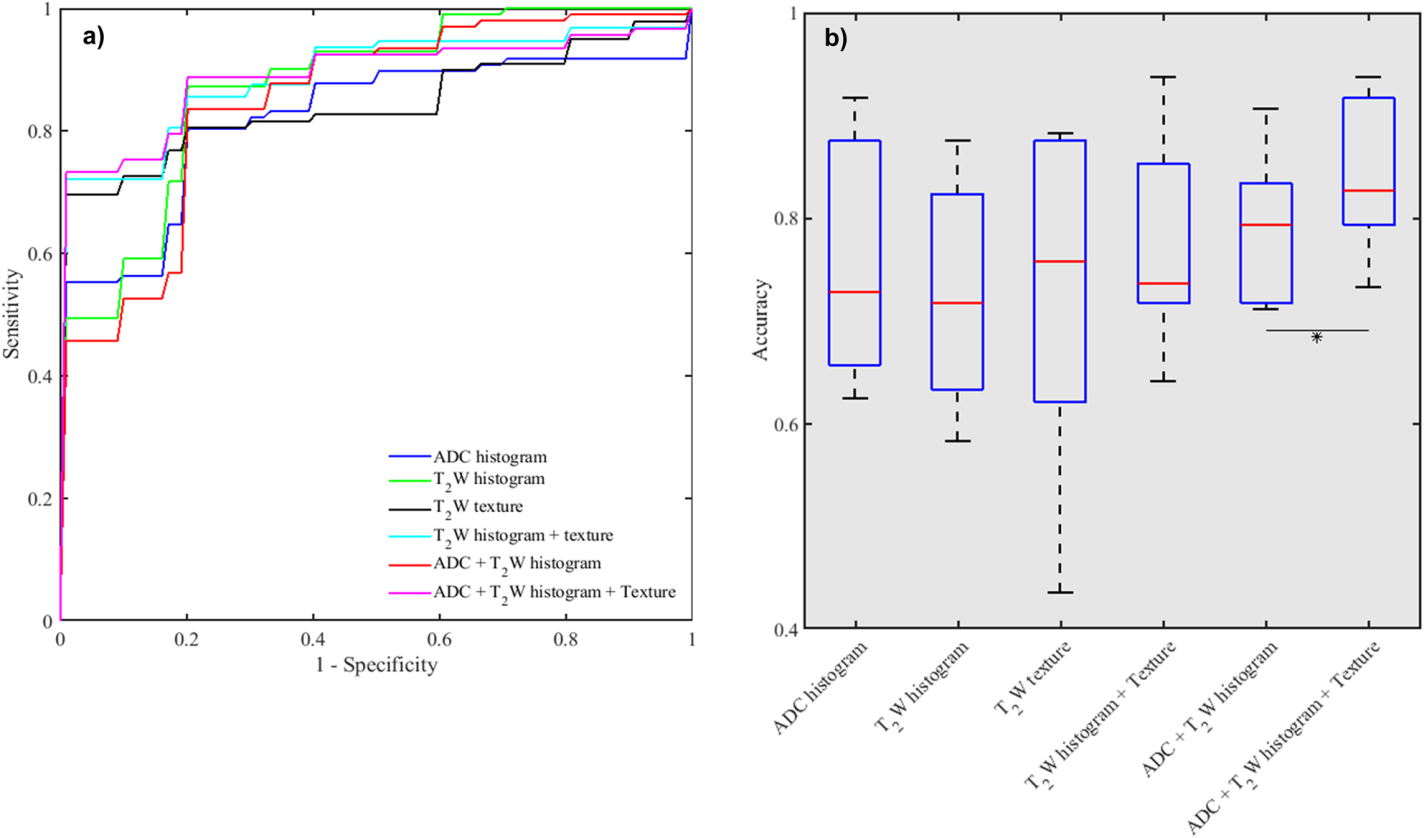
Performance of apparent diffusion coefficient (ADC), T_2_-weighted (T_2_W) histogram and T_2_W textual features in SVM classification of low-aggressive (grade group 1) versus intermediate/high-aggressive (grade group ≥ 2) prostate cancers. (**a**) Receiver operating characteristic curves showing the added value of T_2_W image textural features over traditional intensity histogram features alone. (**b**) Box plot comparing classification accuracies. * indicates significant difference.

Table 3 and Fig. 5 show comparisons of the performance of the feature sets in SVM classification of the two aggressiveness classes at the various institutions. The added value of T_2_W textural features varied generally between the sites. At the individual centers, the augmentation of ADC and T_2_W histogram features with T_2_W textural features resulted in improvement in AUCs at four centers, though not statistically significant (*p* > 0.05). When considering the overall performance across the centers, the differences in AUCs before and after augmentation with textural features were not significant. However, the difference in accuracy was significant (*p* = 0.0218) for ADC + T_2_W histogram versus ADC histogram + T_2_W histogram + T_2_W texture features (Fig. 5). In terms of feature importance within the classifier, a similar trend as in the feature association with cancer grade group was observed. Textural features relating to similarity (grey level non-uniformity), maximum probability, and textural complexity (information measure of correlation) were the most frequently selected, in addition to minimum and 10^th^ percentile from intensity histogram (Table 4).

**Table 3:** Performance of ADC, T_2_W histogram and T_2_W textural features in SVM classification of low-(grade group 1) vs. intermediate/high-aggressive (grade group ≥ 2) prostate cancers at the various institutions.

Feature type	Center for Testing: AUC [95CI] %						Mean [RD]
	A	B	C	D	E	F	
ADC histogram	71 [37–1]	100 [*Nan]	66 [44–88]	88 [73–100]	67 [20–100]	100 [*Nan]	82 [17]
T_2_W histogram	83 [60–100]	100 [*Nan]	86 [70–100]	94 [81–100]	88 [61–100]	73 [39–100]	87 [7]
T_2_W texture	77 [55–99]	100[*Nan]	51[28–74]	96[87–100]	96[84–100]	87[64–100]	84 [16]
T_2_W histogram + Texture	80 [60–100]	94 [*Nan]	76 [57–95]	100 [*Nan]	92 [73–100]	93 [78–100]	89 [8]
ADC + T_2_W histogram	74 [42–100]	94 [*Nan]	78 [57–98]	96 [87–100]	88 [65–100]	80 [47–100]	85 [9]
ADC + T_2_W + Texture	77 [56–99]	94 [*Nan]	75[54–95]	100[*Nan]	96[84–100]	93[78–100]	89 [10]
**Accuracy %**							
ADC histogram	66	88	66	79	63	92	75 [14]
T_2_W histogram	79	88	64	82	58	63	72 [15]
T_2_W texture	62	78	44	88	88	73	72 [18]
T_2_W histogram + Texture	72	94	64	85	75	72	77 [11]
ADC + T_2_W histogram	79	91	71	79	83	72	79 [7]
ADC + T_2_W + Texture	79	94	73	85	92	80	84 [8]
**Sensitivity/specificity %**							
ADC histogram	71/60	75/100	82/50	59/100	75/50	83/100	74/77 [8/30]
T_2_W histogram	79/80	75/100	88/40	65/100	100/17	67/60	79/66 [13/41]
T_2_W texture	64/60	56/100	47/40	76/100	75/100	76/80	64/80 [13/25]
T_2_W histogram + Texture	64/80	88/100	88/40	71/100	100/50	83/60	82/72 [12//30]
ADC + T_2_W histogram	79/80	81/100	82/60	59/100	100/67	83/60	81/78 [10/20]
ADC + T_2_W + Texture	79/80	88/100	76/70	71/100	100/83	100/60	86/82[12/15]

An SVM classifier was trained and tested for each individual institution, where the test data were from the institution being evaluated, and the training data were obtained from the remaining 5 institutions.

*ADC* apparent diffusion coefficient, *T*_*2*_*W* T_2_-weighted, *CI* confidence interval, *SVM* support vector machine, *RD* relative deviation.

*Nan indicates there is no interval.

**Table 4:** Frequency of feature selection for SVM classification of low-(grade group 1) vs. intermediate/high-(grade group ≥ 2) aggressive prostate cancers across six institutions.

Features	Classification model					
	ADC	T_2_W Histogram	T_2_W Texture	T_2_W + Texture	ADC + T_2_W	ADC + T_2_W + Texture
ADC kurtosis	5				5	2
ADC maximum	2				1	0
ADC mean	4				4	1
ADC median	2				3	0
ADC minimum	6				5	3
ADC 10th percentile	4				5	6
ADC 25th percentile	2				5	3
ADC 75th percentile	0				3	1
ADC 90th percentile	3				2	0
ADC Skewness	2				3	0
ADC standard deviation	4				4	2
T_2_W kurtosis		4		1	1	0
T_2_W maximum		6		4	5	0
T_2_W mean		1		1	1	0
T_2_W median		3		1	1	1
T_2_W minimum		6		5	6	6
T_2_W 10th percentile		4		3	4	4
T_2_W 25th percentile		2		1	4	1
T_2_W 75th percentile		3		4	2	0
T_2_W 90th percentile		1		1	0	0
T_2_W skewness		2		2	4	1
T_2_W standard deviation		4		5	4	5
T_2_W ASM			1	3		1
T_2_W Autocorrelation			1	2		0
T_2_W cluster prominence			0	1		0
T_2_W cluster shade			5	2		1
T_2_W contrast			2	2		2
T_2_W correlation			1	1		0
T_2_W difference entropy			2	1		0
T_2_W difference variance			2	3		1
T_2_W dissimilarity			0	2		0
T_2_W entropy			1	1		0
T_2_W IMC 1			6	3		4
T_2_W IMC2			3	3		3
T_2_W IDM			0	1		0
T_2_W maximum probability			2	5		4
T_2_W sum average			0	2		0
T_2_W sum entropy			2	4		1
T_2_W sum variance			0	2		0
T_2_W variance			1	3		0
T_2_W GLNU			5	4		4
T_2_W HGLRE			0	1		0
T_2_W LGLRE			0	2		0
T_2_W long run emphasis			0	2		0
T_2_W long run high GLE			2	3		0
T_2_W long run low GLE			1	3		1
T_2_W RLNU			1	1		1
T_2_W run percentage			0	0		0
T_2_W short run emphasis			0	1		0
T_2_W short run high GLE			0	0		0
T_2_W short run low GLE			1	1		0

*SVM* support vector machine, *ADC* apparent diffusion coefficient, *T*_*2*_*W* T_2_-weighted, *ASM* angular second moment, *IMC* information measure of correlation, *IDM* inverse difference moment, *GLNU* grey level non-uniformity, *HGLRE* high grey level run emphasis, *LGLRE* low grey level run emphasis, *GLE* grey level emphasis, *RLNU* run length non-uniformity.

## Discussion

T_2_W MRI provides high spatial resolution and tissue-specific contrast compared to DW and DCE imaging, but it is predominantly limited to qualitative radiological evaluation of the prostate. In a preliminary study using single-center data^24^, we showed that quantitatively derived T_2_W image textural features have the potential to serve as non-invasive markers for assessing aggressiveness. In this work, we extended and confirmed these findings in a multicenter cohort. T_2_W image textural features, particularly those reflecting homogeneity/similarity (angular second moment, run length non-uniformity, grey level non-uniformity), disorder (entropy) and textural complexity (information measure of correlation) correlated significantly with prostate cancer aggressiveness; and differed significantly between low- and intermediate/high-aggressive prostate cancers as defined by histopathology. Compared to the classifier based on the commonly used histogram metrics from ADC and T_2_W images, the classifier utilizing histogram features augmented with T_2_W textural features performed better, an indication that quantitative texture analysis of anatomical images has the potential to reveal additional morphological and pathophysiological information for radiomics-based assessment of prostate cancer aggressiveness.

The usefulness of entropy/complexity and homogeneity associated textural features in prostate cancer aggressiveness assessment and classification was shown in our previous study^24^, and has also been reported by others^23,36,37^. Histologically, aggressive prostate cancers are characterized by poor differentiation, glandular structure deformation, and loss of cellular integrity of the prostate gland. This disrupts the tissue cyto-architectural patterns, potentially leading to decreased homogeneity and high disorder. Correlations between textural features and prognostic factors and clinical outcome have also been reported^36,38^. If validated, these quantitatively derived T_2_W image features could be combined with other MRI parameters as evidence-based markers for prostate cancer. In the context of this study setup, the findings could particularly be useful in active surveillance situations to follow-up on low-risk cancer patients thereby limiting the need for repeated biopsies.

Although a number of promising studies have reported the utility of MRI texture analysis in prostate cancer^19–23,36–38^, very few are based on multicenter cohorts^22^ or focused on aggressiveness prediction/classification^19,21,23,36^. Multicenter data sharing is important to fulfill the high data demand for training radiomics-based decision support systems. Furthermore, multicenter studies are necessary to ascertain the applicability and robustness of texture analysis and radiomics, and to facilitate their clinical transition across centers. Texture analysis, which considers spatial relationships between pixels rather than individual pixel intensities as in a histogram, could possibly contribute to overcome the inter-institution and scanner variability challenges associated with multicenter data. Compared to DW and DCE imaging, T_2_W imaging is generally regarded as the most stable sequence in terms of imperviousness to scanner variations and gradient artifacts, and tolerance in patients (i.e. contrast agent-free). Although these factors add to its importance, T_2_W imaging is not currently used for quantitative assessment of prostate cancer aggressiveness mainly due to the non-quantitative nature of its signal intensities.

We found the classification performances of the individual feature sets (ADC histogram, T_2_W histogram and T_2_W texture) across the sites to be complementary and in some cases comparable, while the best performance was achieved when the feature sets were combined. The latter observation confirms the preliminary findings from our single-center study^24^. The improved classification performance of T_2_W intensity histogram features compared to some reported studies^19,37^ may be attributable to the post-processing (i.e. intensity non-uniformity correction and standardization) of the T_2_W images, which ensured that the intensities were comparable and had consistent quantitative interpretation across the patients and institutions. Our results are consistent with the findings of previous single-center studies by Fehr et al.^37^ and Chen et al.^19^ who also reported improved characterization of prostate cancer aggressiveness when using combined textural features from ADC and T_2_W images, compared to only mean ADC values or the individual feature sets respectively. On the contrary, Bonekamp et al.^20^ found the performance of mean ADC values to be comparable to that of combined radiomics features from ADC and T_2_W images when classifying benign versus malignant prostate lesions. One possible explanation for this observation is that their study was focused on cancer detection, which could be regarded as a relatively simpler task than aggressiveness classification. Similarly, the aggressiveness dichotomization in our study [i.e. low (grade group 1) vs. intermediate/high (grade group 2–5)] could be considered as a relatively simple classification task compared to for instance grade group 3 versus grade group 4, or the classification of the five distinct groups as in the PROSTATEx-2 Challenge^39^. Surprisingly, in some instances the classifier performance was reduced when textural features were added. Whereas this observation is worth further investigation, we suppose it could be due to potential inefficiencies in the feature selection process.

The goal of this study was to investigate the added value of T_2_W image textural features in prostate cancer aggressiveness assessment with respect to traditional histogram features. Hence, we opted to limit our texture feature extraction to this image sequence even though textural features can also be extracted from ADC maps. Similarly, a wide variety of other radiomic feature types exist that can be extracted including shape, Gabor, wavelet, grey level size zone matrix, neighbouring grey tone difference matrix, etc., features^40,41^. Our choice of using only GLCM and GLRLM features is not based on preference, but, these features have been extensively studied in different tissues and image modalities, and are generally regarded as intuitive. Nevertheless, including additional features may further enhance the classification of prostate cancer aggressiveness.

Feature repeatability and reproducibility are important aspects of radiomics pipeline, especially in a multicenter setting as in this study. Ideally, one would expect a good radiomic feature to be reproducible or stable across centers under the same conditions. A number of studies have recently investigated the repeatability of radiomic features mostly via test–retest analysis^39,42–44^. However, there is no consensus on which feature sets are most repeatable, mainly because the features are heavily influenced by pre-processing configurations during extraction. Most studies^43,44^ show that shape features are more repeatable. In our analysis we found neither interaction between the effects of data origin and aggressiveness nor the main effect of data origin to be significant on any feature, an indication that features were reproducible or stable across the institutions. Despite this, the classification accuracies varied generally within mean relative deviation [7–18%] across the institutions, which may suggest cross-site inconsistency in feature performance. Even though the two statistics (ANOVA & classification) are not directly comparable, a plausible explanation for this latter observation could be due to the highly imbalanced distribution of the data (i.e. classification classes) across the institutions. Even if significant effects were found in the former, it could be due to other potential confounding factors such as inherent differences in the cohort tumor heterogeneity across centers or possible inter-observer differences in the histopathologic grading that was used as ground truth, rather than the features. An alternative and possibly a more robust method of evaluating cross-site reproducibility of radiomics features could be through bootstrapping as employed in the studies by Chirra et al.^39^ and Leo et al. studies^45^, but this method requires a relative higher number of patients per-center.

Our study data and hence methods had some limitations. Despite being a multicenter study, our cohort size is relatively small and the distribution of patients or cancer aggressiveness classes across the centers is imbalanced (Table 1). This could affect our study results, especially the significance of T_2_W textural features in the classification performance. For instance, in a similar classification task performed by Fehr et al.^37^, the added value of textural features was found to be significant only after high imbalances in data was corrected for via sample augmentation. Clinically, prostate cancers are usually categorized into three aggressiveness classes (low, intermediate and high) or more^46^. Due to our study cohort size, however, tumor aggressiveness was stratified into low and intermediate/high. Although this stratification is inadequate for prostate cancer management, it serves as an important basis in the disease management pathway and could also be used as a benchmark for further clinical evaluation in larger multicenter studies. For instance, it is important to rule out low-risk cancers not needing active treatment, which otherwise can be overtreated with associated side effects. The inadequate number of transition zone tumors for analysis limits the applicability of our results to only the peripheral zone. Cancers originating from the transition zone have different radiomic features^22^, they tend to be elusive and are primarily assessed based on their appearance on T_2_W imaging^8^. Hence, the utility of quantitatively derived T_2_W image textural features for transition zone tumor characterization would be of great interest. Machine learning classifier performances are typically evaluated based on their classification accuracy. In this study for instance, the added of value T_2_W textural features can be better appreciated when looking at accuracy scores. Imbalances in datasets that are unrepresentative of the overall population can therefore lead to inflated assessment of the classifier accuracy. However, this was taken into account during the training and prediction (i.e. balanced accuracy) by adjusting the sample weights to be inversely proportional to the class frequencies. Feature repeatability is an important aspect of radiomics pipeline, hence, the lack of this tests in our study is another limitation.

Our study also lacks comparison with clinical readings involving PI-RADS scores, which were not available for this study. However, previous studies^19,47^ have shown radiomics-based models from combined ADC and T_2_W to outperform PI-RADS scores in prostate cancer detection and aggressiveness assessment. Our study setup was based on biopsy proven cases. The performance of textural features in prostate cancer detection and characterization remains to be evaluated prospectively without knowledge of lesion presence and location, especially as textural properties could be affected by inflammation or prostatitis. Also, in future multicenter studies, it would be interesting to perform PI-RADS scoring and VOI segmentation locally at each institution similarly to the histopathology evaluation. Ultimately, further studies in a much larger multicenter cohort are worth investigating.

## Conclusion

This multicenter study confirms that texture analysis of T_2_W images provides quantitative information for assessment of peripheral zone prostate cancer aggressiveness. T_2_W MRI-derived textural features correlated significantly with pathological findings (cancer grade group) from multiple institutions and were sensitive to underlying pathological differences between low- and intermediate/high-grade prostate cancers in the peripheral zone. Although, we found the added value of T_2_W textural features in the classification of these cancer aggressiveness classes to be moderate, our study suggests that T_2_W textural features may have the potential to improve prostate cancer classification in multicenter settings. With a wide array of proposed radiomics features, further studies in larger multicenter cohort would be needed to ascertain their added value and robustness.
